# 5,5′,6,6′-Tetra­methyl-3,3′-bi-1,2,4-tri­azine

**DOI:** 10.1107/S1600536811020691

**Published:** 2011-06-11

**Authors:** Ewa Wolińska, Zbigniew Karczmarzyk, Andrzej Rykowski, Waldemar Wysocki

**Affiliations:** a Department of Chemistry, University of Podlasie, ul. 3 Maja 54, 08-110 Siedlce, Poland

## Abstract

In the title compound, C_10_H_12_N_6_, the two 5,6-dimethyl-1,2,4-triazine halves of the mol­ecule are related by a centre of symmetry. The two triazine rings are coplanar to within a maximum deviation of 0.013 (2) Å from the mean plane of the ring atoms. In the crystal, mol­ecules form layers parallel to the (100) crystallographic plane. Adjacent layers are held together *via* a C—H⋯π inter­action involving mol­ecules related by an *a*-glide plane.

## Related literature

For background information, see: Branowska & Rykowski (2002 ▶); Branowska (2003 ▶); Boger & Weinrab (1987 ▶); Pabst *et al.* (1998 ▶). For the synthesis, see: Dedichen (1936 ▶, 1937 ▶). For a related structure, see: Breu & Range (1993 ▶).
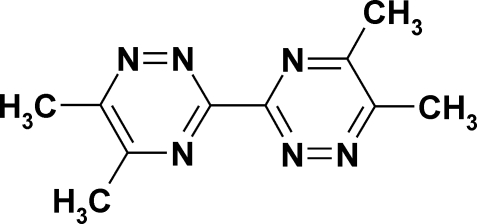


## Experimental

### 

#### Crystal data

C_10_H_12_N_6_
*M* *_r_* = 216.26
Orthorhombic, 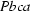

*a* = 8.1167 (7) Å
*b* = 10.6662 (12) Å
*c* = 12.7127 (11) Å
*V* = 1100.59 (18) Å^3^
*Z* = 4
Cu *K*α radiation
μ = 0.71 mm^−1^
*T* = 293 K
0.20 × 0.20 × 0.10 mm

#### Data collection

Kuma KM4 four-circle diffractometer
Absorption correction: ψ scan (North *et al.*, 1968 ▶) *T*_min_ = 0.830, *T*_max_ = 0.929
1637 measured reflections
1205 independent reflections
910 reflections with *I* > 2σ(*I*)
*R*_int_ = 0.100
2 standard reflections every 100 reflections  intensity decay: 1.3%

#### Refinement

*R*[*F* ^2^ > 2σ(*F* ^2^)] = 0.064
*wR*(*F* ^2^) = 0.272
*S* = 1.16
1205 reflections
92 parameters
All H-atom parameters refined
Δρ_max_ = 0.29 e Å^−3^
Δρ_min_ = −0.24 e Å^−3^

### 

Data collection: *KM4B8* (Gałdecki *et al.*, 1996 ▶); cell refinement: *KM4B8*; data reduction: *DATAPROC* (Gałdecki *et al.*, 1995 ▶); program(s) used to solve structure: *SHELXS97* (Sheldrick, 2008 ▶); program(s) used to refine structure: *SHELXL97* (Sheldrick, 2008 ▶); molecular graphics: *ORTEP-3 for Windows* (Farrugia, 1997 ▶); software used to prepare material for publication: *SHELXL97* and *WinGX* (Farrugia, 1999 ▶).

## Supplementary Material

Crystal structure: contains datablock(s) I, global. DOI: 10.1107/S1600536811020691/fy2014sup1.cif

Structure factors: contains datablock(s) I. DOI: 10.1107/S1600536811020691/fy2014Isup2.hkl

Supplementary material file. DOI: 10.1107/S1600536811020691/fy2014Isup3.cml

Additional supplementary materials:  crystallographic information; 3D view; checkCIF report

## Figures and Tables

**Table 1:** Hydrogen-bond geometry (Å, °) *CgA* is the centroid of the triazine ring.

*D*—H⋯*A*	*D*—H	H⋯*A*	*D*⋯*A*	*D*—H⋯*A*
C51—H511⋯*CgA*^i^	1.09 (5)	2.96 (4)	3.616 (3)	119 (3)

Symmetry code: (i) 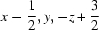.

**Fig. 1.**
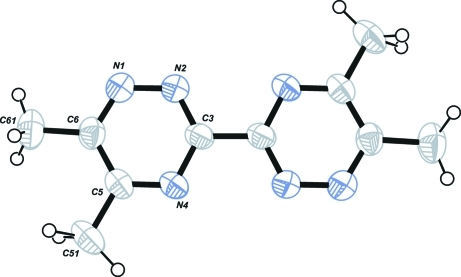
The molecular structure of (I), with atom labels and 50% probability displacement ellipsoids for non-H atoms.

**Fig. 2.**
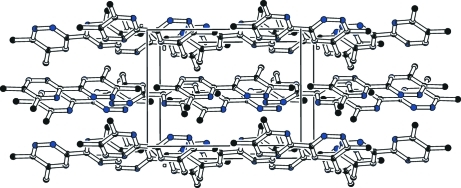
A view of the molecular packing in (I). H atoms are omitted for clarity.

## supplementary crystallographic information

### Comment

In the course of our research we widely explored the synthesis of cycloalkeno[*c*]fused 2,2'-bipyridines using Diels–Alder reactions of 5,5'-bi-1,2,4-triazine derivatives as dienes (Branowska & Rykowski, 2002; Branowska, 2003). However, when we turned our attention to the synthesis of 5,5',6,6'-tetrasubstituted-2,2'-bipyridines, these dienes did not appear useful. Considering the mechanism of the Diels–Alder reaction of 5,5'-bi-1,2,4-triazine, it is clear that to obtain 5,5',6,6'-tetrasubstituted-2,2'-bipyridines, the substituents in positions 5 and 5' of the product have to originate from an unsymmetrical dienofile. Unfortunately, application of such a dienofile can lead to a mixture of 5,5',6,6'- and 3,3',6,6'-tetrasubstituted-2,2'-bipyridines (Boger & Weinrab, 1987). To solve the problem with selectivity, we envisaged that 3,3'-bi-1,2,4-triazines with substituents in 5 and 5' positions can be structurally ideal diene partners in the Diels–Alder synthesis of 5,5',6,6'-tetrasubstituted-2,2'-bipyridines (Pabst *et al.*, 1998). The title compound 5,5',6,6'-tetramethyl-3,3'-bi-1,2,4-triazine was synthesized and its X-ray structure was determined as a part of this research.

The two 5,6-dimethyl-1,2,4-triazine parts of the molecule (I) are related by a crystallographic center of symmetry and possess the *trans* conformation, with the triazine rings being coplanar to within a 0.013 (2) Å maximum deviation from the mean plane. The geometry and conformation of (I) are very similar to those observed in the related structure of 5,5',6,6'-tetraphenyl-3,3'-bi-1,2,4-triazine (Breu & Range, 1993).

In the crystal structure, the molecules of (I) form molecular layers parallel to the (100) crystallographic plane (Fig. 2), with the molecular mean planes being inclined to this plane at an angle of 34.8 (5)°. The layers are held together *via* C—H···π interaction involving the C51—H151 atoms of the methyl group and the triazine ring from the molecule related by an *a*-glide plane.

### Experimental

The title compound, (I), was prepared by the condensation of oxalhydrazidine with 2,3-butanedione according to the procedure of Dedichen (1936, 1937). Crystals suitable for X-ray diffraction analysis were grown by slow evaporation of a benzene solution.

### Refinement

All H atoms were located in a difference Fourier map and their coordinates were refined freely with isotropic displacement parameters *U*_iso_(H) = 1.5*U*_eq_(C). Refined C—H distances were in the range 0.96 (5)–1.09 (5) Å.

### Crystal data

**Table:**

C_10_H_12_N_6_	*D*_x_ = 1.305 Mg m^−^^3^
*M**_r_* = 216.26	Melting point = 441–442 K
Orthorhombic, *P**b**c**a*	Cu *K*α radiation, λ = 1.54178 Å
Hall symbol: -P 2ac 2ab	Cell parameters from 25 reflections
*a* = 8.1167 (7) Å	θ = 11.5–22.4°
*b* = 10.6662 (12) Å	µ = 0.71 mm^−^^1^
*c* = 12.7127 (11) Å	*T* = 293 K
*V* = 1100.59 (18) Å^3^	Prism, yellow
*Z* = 4	0.20 × 0.20 × 0.10 mm
*F*(000) = 456	

### Data collection

**Table:**

Kuma KM4 four-circle diffractometer	910 reflections with *I* > 2σ(*I*)
Radiation source: fine-focus sealed tube	*R*_int_ = 0.100
graphite	θ_max_ = 80.2°, θ_min_ = 7.0°
ω/2θ scans	*h* = −1→10
Absorption correction: ψ scan (North *et al.*, 1968)	*k* = −1→13
*T*_min_ = 0.830, *T*_max_ = 0.929	*l* = −1→16
1637 measured reflections	2 standard reflections every 100 reflections
1205 independent reflections	intensity decay: 1.3%

### Refinement

**Table:**

Refinement on *F*^2^	Secondary atom site location: difference Fourier map
Least-squares matrix: full	Hydrogen site location: difference Fourier map
*R*[*F*^2^ > 2σ(*F*^2^)] = 0.064	All H-atom parameters refined
*wR*(*F*^2^) = 0.272	*w* = 1/[σ^2^(*F*_o_^2^) + (0.2*P*)^2^] where *P* = (*F*_o_^2^ + 2*F*_c_^2^)/3
*S* = 1.16	(Δ/σ)_max_ < 0.001
1205 reflections	Δρ_max_ = 0.29 e Å^−^^3^
92 parameters	Δρ_min_ = −0.24 e Å^−^^3^
0 restraints	Extinction correction: *SHELXL97* (Sheldrick, 2008), Fc^*^=kFc[1+0.001xFc^2^λ^3^/sin(2θ)]^-1/4^
Primary atom site location: structure-invariant direct methods	Extinction coefficient: 0.032 (7)

### Special details

**Table:**

Geometry. All esds (except the esd in the dihedral angle between two l.s. planes) are estimated using the full covariance matrix. The cell esds are taken into account individually in the estimation of esds in distances, angles and torsion angles; correlations between esds in cell parameters are only used when they are defined by crystal symmetry. An approximate (isotropic) treatment of cell esds is used for estimating esds involving l.s. planes.
Refinement. Refinement of F^2^ against ALL reflections. The weighted R-factor wR and goodness of fit S are based on F^2^, conventional R-factors R are based on F, with F set to zero for negative F^2^. The threshold expression of F^2^ > 2sigma(F^2^) is used only for calculating R-factors(gt) etc. and is not relevant to the choice of reflections for refinement. R-factors based on F^2^ are statistically about twice as large as those based on F, and R- factors based on ALL data will be even larger.

### Fractional atomic coordinates and isotropic or equivalent isotropic displacement parameters (Å^2^)

**Table:**

	*x*	*y*	*z*	*U*_iso_*/*U*_eq_	
N1	0.4275 (4)	0.2701 (2)	0.50737 (19)	0.0750 (8)	
N2	0.4248 (3)	0.1532 (2)	0.4693 (2)	0.0733 (8)	
N4	0.58882 (19)	0.08158 (18)	0.61103 (13)	0.0491 (6)	
C3	0.5036 (2)	0.0648 (2)	0.52195 (15)	0.0488 (7)	
C5	0.5959 (2)	0.1973 (2)	0.64669 (16)	0.0501 (7)	
C6	0.5112 (3)	0.2946 (2)	0.59397 (18)	0.0541 (7)	
C51	0.6953 (4)	0.2213 (3)	0.7429 (2)	0.0750 (9)	
H511	0.771 (6)	0.142 (5)	0.767 (3)	0.113*	
H512	0.762 (7)	0.297 (4)	0.732 (3)	0.113*	
H513	0.621 (6)	0.241 (6)	0.803 (3)	0.113*	
C61	0.5097 (4)	0.4263 (3)	0.6326 (3)	0.0733 (9)	
H611	0.482 (6)	0.431 (4)	0.709 (4)	0.110*	
H612	0.427 (5)	0.476 (6)	0.599 (4)	0.110*	
H613	0.617 (5)	0.462 (5)	0.618 (3)	0.110*	

### Atomic displacement parameters (Å^2^)

**Table:**

	*U*^11^	*U*^22^	*U*^33^	*U*^12^	*U*^13^	*U*^23^
N1	0.1042 (18)	0.0492 (13)	0.0717 (14)	0.0018 (11)	−0.0267 (12)	0.0010 (10)
N2	0.1037 (18)	0.0482 (12)	0.0680 (13)	0.0020 (10)	−0.0359 (11)	−0.0007 (9)
N4	0.0483 (9)	0.0560 (12)	0.0431 (9)	−0.0048 (6)	−0.0053 (6)	−0.0018 (6)
C3	0.0512 (10)	0.0529 (13)	0.0424 (10)	−0.0044 (8)	−0.0061 (7)	0.0006 (8)
C5	0.0484 (10)	0.0574 (13)	0.0445 (10)	−0.0097 (7)	0.0017 (7)	−0.0064 (8)
C6	0.0610 (12)	0.0471 (12)	0.0543 (11)	−0.0093 (8)	0.0071 (8)	−0.0029 (8)
C51	0.0804 (16)	0.0834 (19)	0.0613 (14)	−0.0116 (15)	−0.0183 (12)	−0.0177 (13)
C61	0.092 (2)	0.0515 (15)	0.0760 (18)	−0.0118 (12)	0.0119 (14)	−0.0092 (12)

### Geometric parameters (Å, °)

**Table:**

N1—C6	1.320 (3)	C6—C61	1.488 (3)
N1—N2	1.337 (3)	C51—H511	1.09 (5)
N2—C3	1.322 (3)	C51—H512	0.98 (5)
N4—C5	1.316 (3)	C51—H513	1.00 (5)
N4—C3	1.339 (2)	C61—H611	1.00 (5)
C3—C3^i^	1.492 (4)	C61—H612	0.96 (5)
C5—C6	1.414 (4)	C61—H613	0.97 (5)
C5—C51	1.488 (3)		
			
C6—N1—N2	119.7 (2)	C5—C51—H511	114 (2)
C3—N2—N1	118.3 (2)	C5—C51—H512	109 (3)
C5—N4—C3	116.05 (19)	H511—C51—H512	112 (4)
N2—C3—N4	125.6 (2)	C5—C51—H513	110 (3)
N2—C3—C3^i^	116.9 (2)	H511—C51—H513	107 (4)
N4—C3—C3^i^	117.4 (2)	H512—C51—H513	106 (4)
N4—C5—C6	120.27 (19)	C6—C61—H611	112 (3)
N4—C5—C51	118.0 (2)	C6—C61—H612	113 (3)
C6—C5—C51	121.8 (2)	H611—C61—H612	105 (4)
N1—C6—C5	120.0 (2)	C6—C61—H613	108 (3)
N1—C6—C61	117.3 (2)	H611—C61—H613	111 (4)
C5—C6—C61	122.7 (2)	H612—C61—H613	109 (4)
			
C6—N1—N2—C3	1.8 (4)	N2—N1—C6—C5	−0.8 (4)
N1—N2—C3—N4	−0.7 (4)	N2—N1—C6—C61	180.0 (2)
N1—N2—C3—C3^i^	179.2 (2)	N4—C5—C6—N1	−1.4 (3)
C5—N4—C3—N2	−1.4 (3)	C51—C5—C6—N1	178.4 (3)
C5—N4—C3—C3^i^	178.6 (2)	N4—C5—C6—C61	177.8 (2)
C3—N4—C5—C6	2.4 (3)	C51—C5—C6—C61	−2.4 (3)
C3—N4—C5—C51	−177.4 (2)		

Symmetry codes: (i) −*x*+1, −*y*, −*z*+1.

### Hydrogen-bond geometry (Å, °)

**Table:**

CgA is the centroid of the triazine ring.

**Table:**

*D*—H···*A*	*D*—H	H···*A*	*D*···*A*	*D*—H···*A*
C51—H511···CgA^ii^	1.09 (5)	2.96 (4)	3.616 (3)	119 (3)

Symmetry codes: (ii) *x*−1/2, *y*, −*z*+3/2.
